# Tetragonal BaCoO_**3**_: A Co^**4+**^ Ferromagnetic Mott Insulator

**DOI:** 10.1021/acs.jpcc.5c03983

**Published:** 2025-10-23

**Authors:** Mingyu Xu, Haozhe Wang, Krishna Prasad Koirala, Corey Melnick, Cheng Peng, Mario U. González-Rivas, Jiaqi Lu, Le Wang, Jessica Freese, Mark H. Engelhard, Yingge Du, Xianglin Ke, Robert J. Green, Alannah M. Hallas, Jie Li, Gabriel Kotliar, Weiwei Xie

**Affiliations:** † Department of Chemistry, 3078 Michigan State University, East Lansing, Michigan 48824, United States; ‡ Physical and Computational Sciences Directorate, 6865 Pacific Northwest National Laboratory, Richland, Washington 99354, United States; § Condensed Matter Physics and Materials Science Department, 330908 Brookhaven National Laboratory, Upton, New York 11973, United States; ∥ Department of Physics & Astronomy, 8166 University of British Columbia, Vancouver, British Columbia V6t 1z1, Canada; ⊥ Stewart Blusson Quantum Matter Institute, 7235 University of British Columbia, Vancouver, British Columbia V6t 1z4, Canada; # Department of Earth and Environmental Sciences, 1259 University of Michigan, Ann Arbor, Michigan 48109, United States; ∇ Department of Physics and Engineering Physics, University of Saskatchewan, Saskatoon S7n 5e2, Saskatchewan, Canada; ○ Energy and Environment Directorate, 6865 Pacific Northwest National Laboratory, Richland, Washington 99354, United States; ◆ Department of Physics and Astronomy, 3078 Michigan State University, East Lansing, Michigan 48824, United States; ¶ Department of Physics and Astronomy, Rutgers University-New Brunswick, Piscataway, New Jersey 08854, United States

## Abstract

We report the stabilization of the metastable body-centered tetragonal (BCT) phase of BaCoO_3_ (BCT-BaCoO_3_) under high-pressure (15 GPa) and high-temperature (1200 °C) conditions using a mixture precursor. This double perovskite adopts the EuTiO_3_-type structure (space group *I*4/*mcm*), as confirmed by powder X-ray diffraction and high-resolution STEM. X-ray photoelectron spectroscopy indicates a predominant Co^4+^ oxidation state without detectable oxygen vacancies. Magnetization and heat capacity measurements reveal ferromagnetic ordering at *T*_C_ ∼ 107 K, attributable to the BCT-BaCoO_3_ phase. Above this temperature, the mixed-phase sample exhibits Curie–Weiss paramagnetism, a low-spin to high-spin crossover upon cooling, and a possible intermediate-spin state at elevated temperatures. Resistivity data indicates insulating behavior with weak magnetoresistance. DFT and DFT + DMFT calculations suggest that the insulating state originates from an orbitally selective transition sensitive to the nominal valence of the Co-d shell. The metastable BCT-BaCoO_3_ phase cannot be retained in pure form at ambient pressure but can be stabilized by embedding it in a disordered mixture, offering a potential route to discover and preserve other high-pressure phases under ambient conditions.

## Graphical abstract

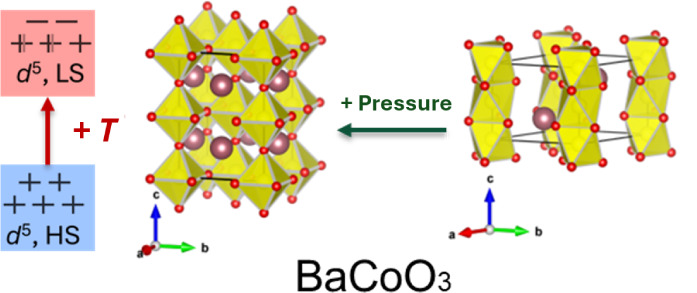

## Introduction

Transition metal oxides with perovskite crystal structures exhibit a broad spectrum of magnetic and electronic phenomena, including high-temperature superconductivity,^1,2^ colossal magnetoresistance,^3^ ferroelectricity,^4^ as well as metal-to-insulator, structural, and magnetic phase transitions. These perovskites, with the general formula ABO_3_, can adopt either a three-dimensional framework of corner-sharing [BO_6_] octahedra (in cubic (C) or pseudocubic forms) or one-dimensional chains of face-sharing [BO_6_] octahedra in hexagonal (H) arrangements.^5^ Cobalt-based perovskites (B = Co), such as the hexagonal 2H-BaCoO_3_ ^6,7^ and cubic SrCoO_3_,^8^ exemplify this diversity. In 2H-BaCoO_3_, face-sharing [CoO_6_] octahedra form parallel chains along the *c*-axis, creating a two-dimensional triangular lattice in the *ab*-plane with a Co^4+^–O^2–^–Co^4+^ angle of approximately 78°.^7^ This structure contrasts with SrCoO_3_, where the Co^4+^ ions are linked in three equivalent directions by 180° Co^4+^–O^2–^–Co^4+^.^8^ Substituting Sr^2+^ and Ba^2+^ with smaller Ca^2+^ ions leads to Ca_3_Co_2_O_6_ in the Sr_4_PtO_6_-type structure, diverging from the typical ABO_3_ structure.^9^ The orthorhombic GdFeO_3_-type structure of CaCoO_3_ is stabilized exclusively through high-pressure oxygen annealing,^10^ showcasing the structural complexity of these materials.

Cobalt-based perovskites have garnered significant interest for their spin-state transitions and unusual Co spin states observable within experimentally accessible temperature ranges.^11^ This is attributed to the comparable magnitudes of the crystal electric field (CEF) splitting of Co d states and Hund’s rule exchange energy.^12^ The small energy gap between the *t*_2g_ and *e*_g_ states enables the thermal excitation of *t*_2g_ electrons to *e*_g_ states, resulting in higher spin states.^13,14^ Spin-state transitions have been reported in LaCoO_3_,^15,16^ Pr_1–*x*_Ca_*x*_CoO_3_,^17^ and GdBaCo_2_O_5.5_,^18^ with intermediate spin states observed in LaCoO_3_ ^19^ and La_1–*x*_Sr_*x*_CoO_3_.^20,21^ Conversely, 2H-BaCoO_3_ exhibits weak magnetism, with its high-temperature magnetic susceptibility suggesting the presence of low-spin *S* = 1/2 Co^4+^. In contrast, SrCoO_3_, a ferromagnetic (FM) metal with a Curie temperature *T*_CW_ of ∼280 K,^22^ showcases the complexity due to Co^4+^’s nominally d^5^ character (yielding spin-1/2 and quantum effects), strong O p–Co d orbital hybridization, and spin–orbit coupling (SOC).^23^ The origin of the near-room-temperature ferromagnetism in cubic perovskite SrCoO_3_ has been the subject of extensive discussion.^24^ Further reducing the size of the A-site cation, causing significant tilting of [CoO_6_] octahedra and narrowing of the electronic bandwidth, results in CaCoO_3_ exhibiting antiferromagnetic (AFM) ordering at 95 K with a probable helical spin arrangement, while it remains in an incoherent metallic state even at low temperatures.^25^

The dependence of magnetism on A-site cation size in ACoO_3_ perovskites inspired our high-pressure investigation of BaCoO_3_ to tune lattice parameters and crystal structures without introducing new elements. We recently reported that BaCoO_3_ retains its 2H-type crystal structure with a shorter Co–Co distance of approximately 2.07 Å, synthesized at 6 GPa and 1200 °C for 3 h.^7^ The potential for achieving even shorter Co–Co distances, possibly inducing complex magnetism and unusual Co spin states, motivated further exploration at higher pressures. Here, we discovered that treatment at 15 GPa and 1200 °C for 3 h transforms 2H-BaCoO_3_ into a tetragonal symmetric perovskite phase, termed as BCT-BaCoO_3_.

## Methods

### High Pressure and High Temperature Synthesis

The synthesis was conducted using a 1000-ton multianvil apparatus (MA) at the University of Michigan. The starting material was synthesized at ambient pressure, which was prepared by thoroughly mixing the BaCO_3_ and CoO in the atomic ratio of 1:1 and subsequently heating at 1100 °C for 72 h before quenching. The sample was kept at 110 °C overnight to remove the moisture before loading for high-pressure synthesis. Pure BaCoO_3_(P6_3_/*mmc*) is prepared at 800 °C for 72 h before quenching.^26^ The sample was kept at 110 °C overnight to remove the moisture before loading for high-pressure synthesis. Pure BaCoO_3_(P6_3_/*mmc*) is prepared at 800 °C for 72 h before quenching. The COMPRES 10/5 cell assemblies were used in the synthesis.^27^ The sample was loaded in a platinum capsule and kept at 15 GPa and 1200 °C for 3 h before quenching to room temperature and then decompressed to ambient pressure. High-pressure and high-temperature synthesis were processed three times to get consistent results. To clarify the stabilization pathway, we directly compared the two precursor routes. When a pure 2H-BaCoO_3_ precursor annealed at 800 °C was subjected to high-pressure synthesis, the product reverted to the hexagonal phase upon decompression, with no evidence of the metastable tetragonal BCT-BaCoO_3_ phase being retained (Figures S10–S12). In contrast, the precursor with some impurity of BaCoO_*x*_ annealed at 1100 °C yields BCT-BaCoO_3_, which is reproducibly stabilized at ambient pressure (Figures S4–S6).

### Phase Analysis

The phase identity and purity were examined using a Bruker Davinci powder X-ray diffractometer with Cu K_α_ radiation (λ = 1.5406 Å). Samples were ground using a mortar and pestle at room temperature, and then the powder was put on the vacuum grease-coated silicon puck. Room temperature measurements were carefully performed with a step size of 0.010° at a scan speed of 5.00 s/step over a Bragg angle (2θ) range of 15–90°. The synthesis product was examined using the JEOL-7800FLV field emission SEM at the Robert B. Mitchell Electron Microbeam Analysis Lab (EMAL) of the University of Michigan, and the analyses confirmed chemical purity and homogeneity.

### Structure and Chemical Composition Determination

The structural and chemical composition determination was conducted using high-angle annular dark field (HAADF) imaging and energy dispersive X-ray spectroscopy (EDS) inside a scanning transmission electron microscope (STEM). The TEM sample was prepared by using a dual beam Helios instrument, which combines focused ion beam (FIB) and scanning electron microscopy. First, a cross-sectional lamella was extracted from the polycrystalline sample using FIB milling. The lamella gradually thinned down to approximately 200 nm at 30 kV. Subsequently, the sample was further reduced to thickness to around 50 nm at 5 kV. The final polishing of the sample was carried out at 2 kV. For HAADF imaging, a Themis-Z STEM microscope equipped with an aberration corrector and a monochromator was used. An acceleration voltage of 300 kV and a prove current of approximately 30 pA were employed for both STEM imaging and EDS mapping. In HAADF imaging, a convergence angle of 30 mrad and collection angle of 60 to 180 mrad were used.

### Chemical Valence State Analysis

X-ray photoelectron spectroscopy (XPS) measurements were performed using a Thermo Fisher NEXSA spectrometer with a 125 mm mean radius, full 180° hemispherical analyzer, and 128-channel detector. This system uses a focused monochromatic Al K_α_ X-ray (1486.7 eV) source for excitation and an electron emission angle of 60 degrees. The narrow scan spectra were collected using a pass-energy of 50 eV with a step size of 0.1 eV. For the Ag 3d_5/2_ line, these conditions produced a fwhm of 0.84 eV ± 0.02 eV. The binding energy (BE) scale is calibrated using the Cu 2p_3/2_ feature at 932.62 ± 0.05 eV and Au 4f_7/2_ at 83.96 ± 0.05 eV.

### Physical Properties Measurement

Magnetization measurements were carried out using a Quantum Design MPMS 3 magnetometer and PPMS after demagnetization using SQUID and VSM. Sample are glued to the no-background quartz rod with GE varnish. Temperature dependent magnetization was measured over the temperature range of 2–300 K employing the zero-field cooled (ZFC) and field cooled (FC) protocols. Magnetic hysteresis loops were recorded with applied fields up to 7 T. Electrical resistivity measurements were conducted with four-probe methods using platinum wires on a polycrystalline sample of BCT-BaCoO_3_ in the dimensions of 1.0 × 0.8 × 1.0 mm^3^ with a Quantum Design physical property measurement system (PPMS) DynaCool.

### Electronic Structure Calculation

We conduct all-electron density functional theory (DFT) and charge self-consistent DFT with dynamical mean field theory (DFT + DMFT) of BCT-BaCoO_3_ using *Portobello*.^28–30^ The DFT equations are solved within the generalized-gradient approximation (GGA) using the Perdew–Burke–Ernzerhof (PBE)^31^ functional, neglecting the spin–orbit coupling. An 8 × 8 × 8 *k*-mesh and basis with RK of 8 are used for all calculations. The DMFT equations are used to treat correlations within the Co d-shell, where the off-diagonal elements in the Hamiltonian are truncated in order to avoid a sign problem during the solution of the quantum impurity problem. We use a spherically symmetric Slater–Condon interaction^32^ with Hubbard *U* = 10 eV and Hund *J* = 1 eV to describe the interaction, and we use the fully localized limit double-counting with an electron occupancy of *N*_0_ = 5, which corresponds to the nominal *d* ^5^ valence.

### X-ray Absorption Spectroscopy

X-ray absorption spectroscopy (XAS) experiments were carried out at the REIXS beamline of the Canadian Light Source.^33^ The experiment was carried out at normal incidence, at several temperatures between 20 and 300 K, spanning the different regions identified from the Curie–Weiss fit of the magnetic susceptibility. Total electron yield was obtained from by measuring the drain current from the sample. The samples were mounted on silver paint to improve thermal contact. Data collection was carried out between 750 and 830 eV, spanning Co L2,3 and Ba M4,5 resonances. Due to the overlap between the Co and Ba transitions, a BaTiO_3_ thin film was used as a Ba^2+^ reference. The BaTiO_3_ spectrum was used to remove the Ba M4,5 white line from the BaCoO_3_ sample.

## Results and Discussion

The sample was synthesized under high-pressure conditions (15 GPa) using a multianvil apparatus. However, due to the limited quantity and the metastable phase of the final product at ambient pressure, we can only obtain limited-quality powder X-ray diffraction results. Even accounting for the potential phase transition that may have occurred during the grinding of the metastable phase at ambient pressure, there is clear evidence of a structural change from *P*6_3_/*mmc* to *I*4/*mcm* as shown in Figure S4a. We also investigated the crystallographic properties of BCT-BaCoO_3_ using atomically resolved high-angle annular dark field (HAADF) and integrated differential phase contrast (iDPC) imaging techniques in a scanning transmission electron microscope (STEM). Figure 1a presents the body-centered tetragonal perovskite unit cell. Figure 1b, a representative HAADF-STEM image acquired along the *b*-axis, confirms BCT-BaCoO_3_’s high crystallinity over a large area. Figure 1c presents the Fast Fourier Transform (FFT) of the HAADF-STEM image. The diffraction spot arrangement in the FFT matches well with the tetragonal double perovskite EuTiO_3_-type structure (*I*4/*mcm*, No. 140). Notably, we observed an *n*-layer twin feature on the (−202) plane, indicating a superstructure phase. Such twin features typically arise from thermal stress applied during crystal growth.^34^ Figure 1d offers atomically resolved HAADF images, clearly visualizing Ba and Co atom positions. Figure 1e highlights and overlays these positions on the images. Figure 1f, an iDPC image, shows the tetragonal structure, including the arrangement of oxygen atoms. Energy dispersive X-ray spectroscopy (EDS) mapping, shown in Figure S1, confirmed the uniform distribution of elements in BCT-BaCoO_3_. EDS analysis, summarizing the concentration variations of Ba, Co, and O atoms, is presented in Figure S2 and Table S1. Significantly, within the margin of error, no detectable oxygen vacancies were observed. Powder X-ray diffraction is refined with the CIF obtained from STEM, shown in Figure S4.

**Figure 1:**
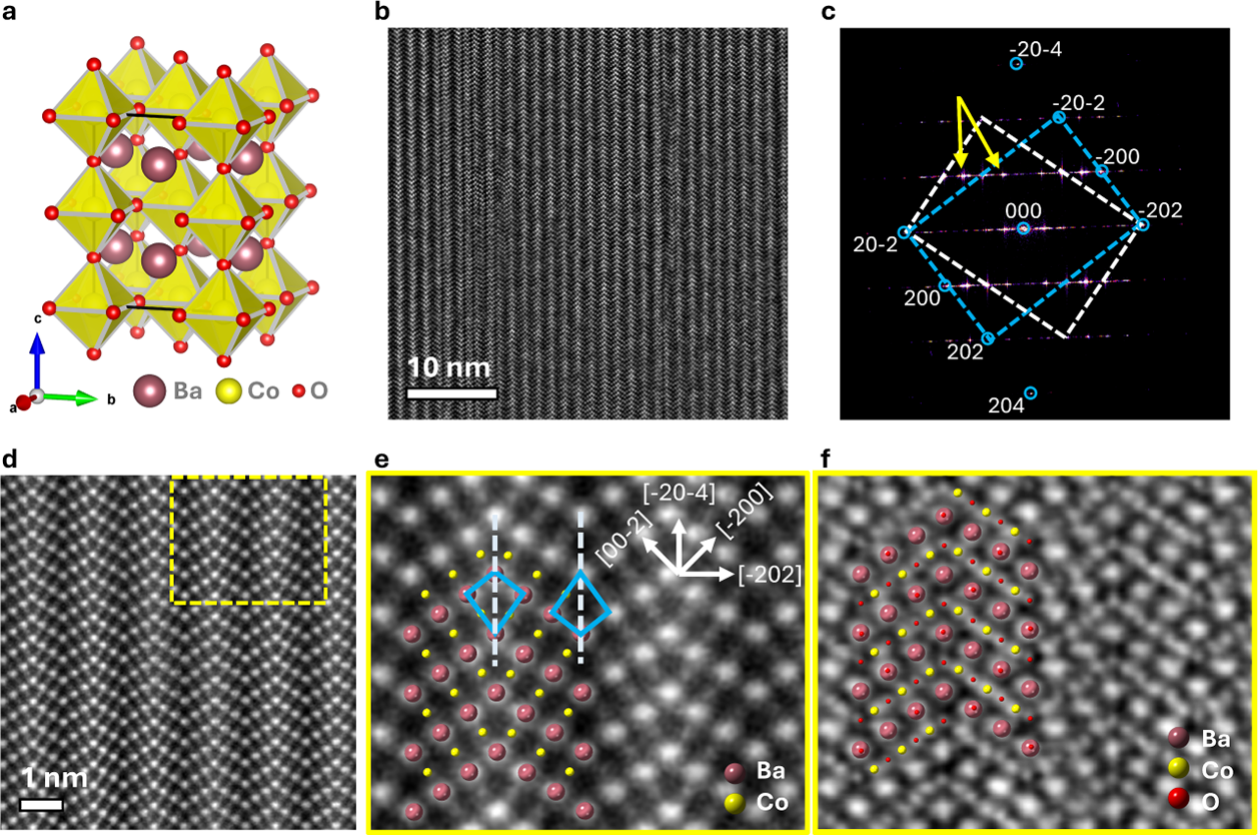
Crystal structure determination of BCT-BaCoO_3_. a, Unit cell of the tetragonal BCT-BaCoO_3_ phase (*I*4/*mcm*), with Ba, Co, and O shown in brown, yellow, and red, respectively. b, Representative HAADF-STEM image viewed along the [010] zone axis (scale bar = 10 nm). The n-layer twin structure along the (−202) plane is explicitly marked with dashed lines. c, Fast Fourier Transform (FFT) of the HAADF image in Figure 1 *b*, showing diffraction spots consistent with the tetragonal EuTiO_3_-type structure; additional spots arising from the periodic twins are indicated by yellow arrows. Zone axis is annotated. d, Atomically resolved HAADF image (scale bar = 1 nm), highlighting the ordered arrangement of Ba and Co atoms. e, Magnified region from panel d overlaid with the *I*4/*mcm* structural model, where the twin boundaries along (−202) are outlined. f, iDPC-STEM image (scale bar = 1 nm) viewed along [010], overlaid with the tetragonal structure, showing both cation and oxygen positions.

Figure 2 compares the unit cells and structures of BCT-BaCoO_3_, 2H-BaCoO_3_, SrCoO_3_, and CaCoO_3_. In BCT-BaCoO_3_, corner-sharing [CoO_6_] octahedra form a 3D network, with Ba atoms occupying cuboctahedra cavities in a perovskite structure. Co–O–Co angles are 180° along the *c*-axis and approximately 173° in the *ab*-plane, attributed to the slight rotation of [CoO_6_] octahedra parallel to the *c*-axis. The Co–O bond lengths in BCT-BaCoO_3_ are 1.95 Å, varying slightly, compared to 1.892(1) Å in 2H-BaCoO_3_. Consequently, [CoO_6_] octahedra in BCT-BaCoO_3_ are larger than in the ambient pressure phase 2H-BaCoO_3_, with the expected increase in density from hexagonal to (pseudo) cubic BaCoO_3_. The low atomic packing density and close Co–Co distances in 2H-BaCoO_3_ likely induce the observed structure transition, lowering the system’s total energy in response to external pressure.

**Figure 2:**
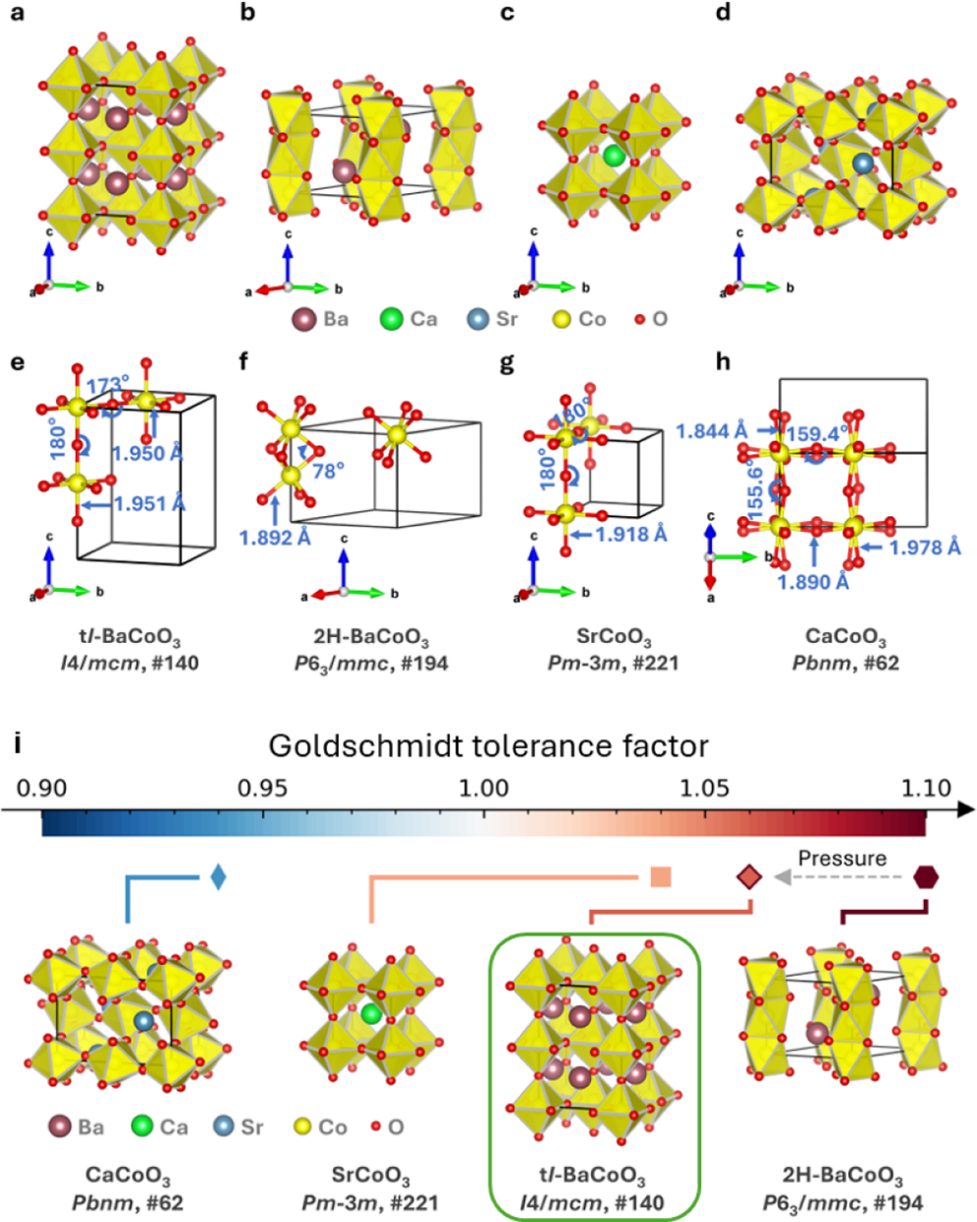
Crystal structure comparisons of BCT-BaCoO_3_, 2H-BaCoO_3_, SrCoO_3_, and CaCoO_3_. a–d, Unit cell of BCT-BaCoO_3_ (*I*4/*mcm*, No.140), 2H-BaCoO_3_ (*P*6_3_/*mmc*, No.194), SrCoO_3_ (*Pm*-3*m*, No.221), and CaCoO_3_ (*Pbnm*, No.62). Brown, green, blue, yellow, and red indicate Ba, Sr, Ca, Co, and O atoms, respectively. e–h, Comparison of Co–O bond lengths and Co–O–Co bond angles, with an emphasis on structural anisotropy in these structures. i, Goldschmidt tolerance factors and the structural stability of BCT-BaCoO_3_, 2H-BaCoO_3_, SrCoO_3_, and CaCoO_3_.

Structure stability and diversity in ACoO_3_ can be manipulated through Goldschmidt tolerance factor by modifying the atomic size on A-site. Unlike BaCoO_3_, SrCoO_3_ with the smaller Sr cation occupying the A-site exhibits a CaTiO_3_-type cubic structure (*Pm*-3*m*, No. 221) with regular octahedra forming a 3D network and Co–O bond lengths of 1.918 Å. The replacement of the A-site with an even smaller cation in CaCoO_3_ results in an orthorhombic GdFeO_3_-type structure, with similar *a* and *c* axes resulting from [CoO_6_] octahedra distortion and rotation, leading to Co–O bond lengths of 1.844(1) and 1.978(1) Å. Although the phase was synthesized under high-pressure and high-temperature conditions, the atomic radius correlations still conform to the Goldschmidt tolerance factor rule in Figure 2i.

To investigate the magnetic properties of BCT-BaCoO_3_, we measured temperature-dependent magnetization in zero-field cooled (ZFC), field cooled (FC) modes at 10 and 100 Oe, as illustrated in Figure 3a, and field cooled up to 90 kOe using a repeatedly synthesized sample shown in Figure S5. Since the sample contains multiple phases, as indicated in Figure S4, it is proposed that the magnetization originates from the average formula BaCoO_3_, rather than only from the new BCT-BaCoO_3_ phase, however, based on the 50 K shift of transition temperature after high pressure and high temperature treatment, the magnetization signals should mainly come from BCT-BaCoO_3_. Magnetization and heat capacity data will be corrected to the proper ratio of BCT-BaCoO_3_ when describing the properties of BCT-BaCoO_3._ A ferromagnetic transition (FM) was identified at *T*_C_ = 107 K, indicated by the sharp increase in the susceptibility, while the ordering temperature was defined by taking the minimum in the temperature derivative of χ*T*, depicted in Figure 3b, and also the feature in heat capacity measurement shown in Figure S6. Below the FM ordering temperature, the magnetic susceptibility exhibits splitting between ZFC and FC modes, suggesting a relatively hard ferromagnetic state with significant hysteresis. This transition should come from BCT-BaCoO_3_, since other phases do not have the ferromagnetic transition above 80 K. Magnetization comparison before and after high-pressure and high-temperature treatment is shown in Figure S5. Compared to ambient pressure 2H-BaCoO_3_, BCT-BaCoO_3_ shows a marked enhancement in FM interactions, likely due to changes in the Co–O–Co bond angle. This is crucial for determining magnetic superexchange interactions that significantly affect magnetic behavior.^35^

**Figure 3:**
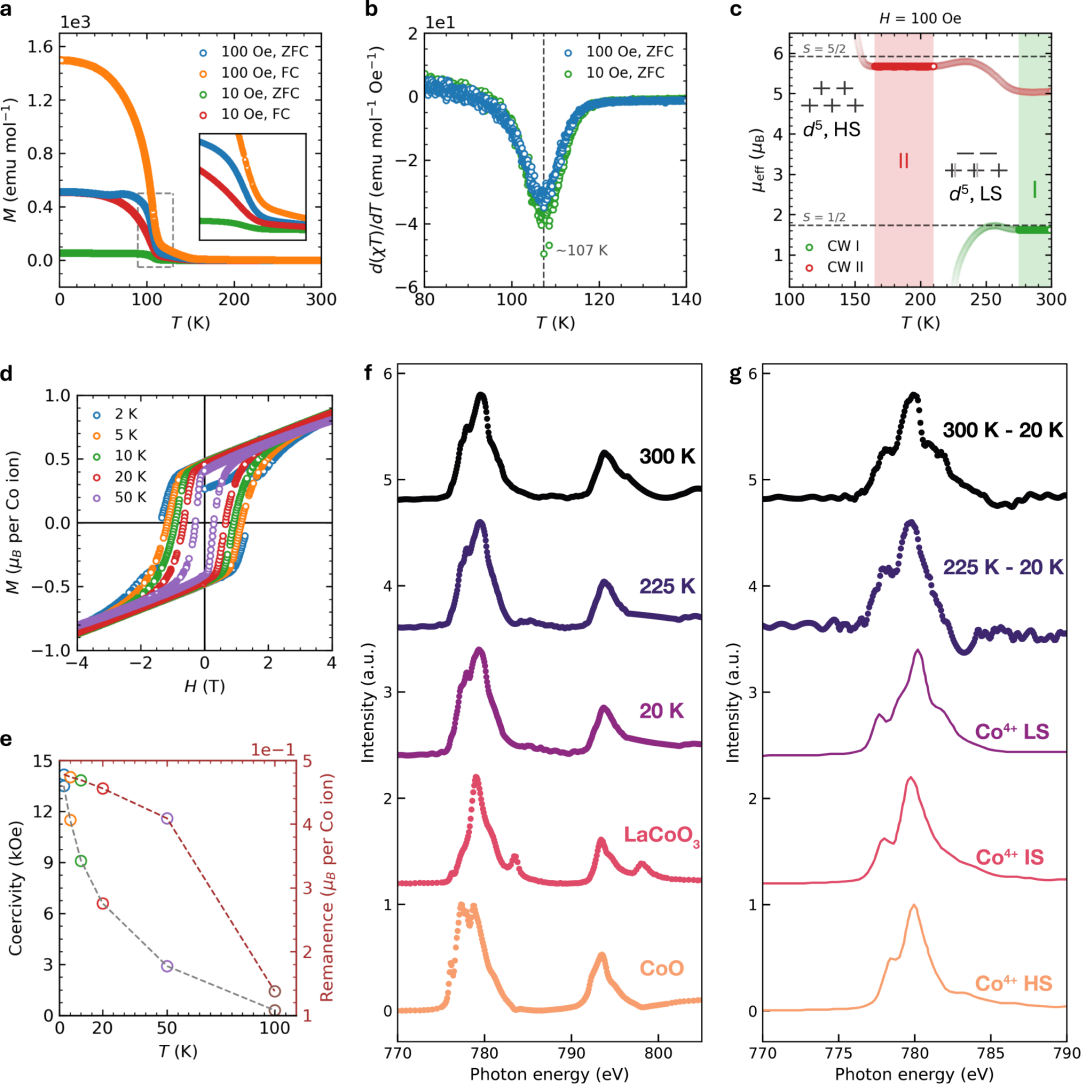
Temperature-dependent magnetization of the mixed phases containing BCT-BaCoO_3_. a, Magnetic susceptibility as a function of temperature at 10 and 100 Oe, in ZFC and FC modes. Inset: Detailed view of the area within the gray dashed box, near the FM ordering temperature. b, Temperature derivative of χ*T*, indicating the ferromagnetic ordering around 107 K, as suggested by the minimum. c, Magnetic susceptibility in temperature Regions I and II, analyzed using the Curie–Weiss law. Regions I and II are depicted in green and red, respectively. d, Field-dependent magnetization at different temperatures. The mixture sample is considered to contain average BsCoO_3_ formula. e, Temperature-dependent coercivity and remanence increase as temperature decreases. f, XAS spectra for the mixture phase containing BCT-BaCoO_3_ collected at 300, 225, and 20 K and two standard materials, LaCoO_3_ and CoO. g, XAS difference spectra (300 K–20 K and 225 K–20 K) and Co^4+^ multiplet calculations for different possible spin states.

Near room temperature, this mixed material predominantly exhibits Curie–Weiss paramagnetism. Figure S6 presents the inverse magnetic susceptibility above the FM transition temperature, revealing complex magnetic behavior. Within this temperature range, we identify two distinct temperature regions: 165–210 K (Region I) and 275–300 K (Region II) before the phase transition. In Region I, the magnetic susceptibility data were fitted with the modified Curie–Weiss law (eq 1),

1 $$\chi =\chi_{0}+\frac{C}{T-\theta_{\mathrm{CW}}}$$

where θ_CW_ is the Curie–Weiss temperature, χ_0_ is the temperature-independent susceptibility, and *C* is the Curie constant. The fit yielded a θ_CW_ of 225.6(4) K and a μ_eff_ of 1.62(1) μ_B_ per formula, as detailed in Figure S7. The μ_eff_ value closely approximates the calculated spin-only moment of 1.73 μ_B_ for low-spin (LS) Co^4+^ (d^5^ electron configuration, *S* = 1/2). In Region II, Curie–Weiss behavior is still observed, shown in Figure S7, providing θ_CW_ of 97.8(2) K and μ_eff_ of 5.67(1) μ_B_ per formula, in which the effective magnetic moment is much larger than Region I. We note that the spin-only moment for high-spin (HS) Co^4+^ with *S* = 5/2 is 5.92 μ_B_. Consequently, a temperature-induced crossover between LS and HS configurations in this system may be attributed to comparable levels of crystal electric field (CEF) splitting (Δ_CEF_) and Hund’s rule exchange energy (*J*_ex_). Unlike other spin state transitions where LS state could be thermally activated to HS state, this HS–LS crossover indicates that in this mixture system, Δ_CEF_ is unusually smaller than *J*_ex_. Together with the structure information displayed in Figure 1, we would assign this as a result of the longer Co–O bond length in [CoO_6_] octahedra compared to 2H-BaCoO_3_, which reduces electron repulsion and then gives a smaller Δ_CEF_. This crossover is more evident in Figure 3c, where μ_eff_ values are plotted against temperature using two Curie–Weiss models. Between Region I and II, we hypothesize that either an intermediate-spin (IS) state (*S* = 3/2) emerges or the system behaves as a mix of LS and HS states, similar to LaCoO_3_.^16^ These spin states may coincide with a second-order structural phase transition. However, we assume no tetragonal elongation or compression of [CoO_6_] octahedra in Region I, with Co *d* states split in a standard octahedral field, indicating an absence of Jahn–Teller effect (JTE). In fact, for the d^5^ electron configuration, JTE will not create an energy difference for the HS state, and only weak JTE is possible for the LS state as well as the IS state because of the unevenly occupied *t*_2g_ orbitals. Upon further cooling from Region II, ferromagnetic interactions predominate, leading to a breakdown of Curie–Weiss behavior.

The field-dependent magnetization of the mixture phase, illustrated in Figure 3d, was measured. Figure 3d displays the hysteresis loop observed below the FM ordering temperature, notably below 50 K. Coercivity and remanence increase as the temperature decreases, reaching maximum values of approximately 13.5 kOe and 0.48 μ_B_, respectively, at 2 K, as demonstrated in Figure 3e. High coercivity at low temperatures indicates hard ferromagnetism within the system. Magnetization did not reach full saturation up to 7 T, where the magnetic moment measured approximately 1.09 μ_B_/Co at 2 K (see Figure S8). This observed moment, suggestive of an unsaturated *S* = 5/2 HS Co^4+^ state, provides further evidence for the LS–HS crossover upon cooling, just above the FM ordering temperature. Figure S8 illustrates field-dependent magnetization above 100 K, with negligible hysteresis observed. Linear responses at 200 and 300 K confirm the paramagnetic behavior at high temperatures.

X-ray absorption spectroscopy (XAS) measurements allow us to directly probe unoccupied Co *d* orbitals in BaCoO_3_ mixture and then determine their spin state. Normalized XAS data for the mixed phases and Co^2+^ (CoO) and Co^3+^ (LaCoO_3_) standards are presented in Figure 3f. The BaTiO_3_ thin film was used as a Ba^2+^ reference. While this subtraction procedure is widely adopted, the main source of uncertainty arises from small differences in normalization and energy alignment between the reference and sample spectra. No standard is available for Co^4+^. However, we can reliably distinguish it from Co^2+^ and Co^3+^ by the shift in the centroid toward higher energies. Representative data sets are shown above (300 K) and below (225 K) its low spin to high spin crossover and within its magnetically ordered state (20 K). For the mixed phases containing BCT-BaCoO_3_, all spectra are mainly assigned to Co^4+^, as is evident from a shift of the spectra’ centroids to higher energies compared to the 2+ and 3+ references. However, we also observe fractions of Co^2+^ and Co^3+^, which can be identified from small inflections in the rising edge. The spectra presented here were collected in total electron yield (TEY) mode, which is known to be surface sensitive, suggesting these extra contributions are surface charge states, compensating for the rather unstable ligand-hole nature of the Co^4+^ state. However, the Co^4+^ signals observed here can still be expected to represent the bulk behavior of this species in the sample. Direct assignment of the spin state from the different temperatures’ XAS is not possible due to the presence of various contributing signals (Co in 2+, 3+, and 4+ states). However, the change in spin state can still be inferred by monitoring the change in the difference spectra obtained by subtracting the 20 K signal from the higher temperature spectra. These are shown (normalized) in Figure 3g along with reproduced representative ligand field multiplet theory calculations for the low, intermediate, and high spin states of Co^4+^.^36^ Most notably, the shoulder at 782 eV on the right-hand side of the central absorption peak in the 300–20 K difference trace is in good agreement with only the spectra of Co^4+^ LS, while that shoulder is strongly suppressed in the 225–20 K difference trace. The XAS, therefore, corroborates an electronic transition from Co^4+^ LS to HS. Since only TEY mode was available for the small metastable samples, FY-XAS could not be obtained. Therefore, the conclusions are based on surface-sensitive TEY data and should be interpreted as consistent with, rather than definitive proof of, the spin-state evolution.

The electronic properties of cobalt oxides are inherently linked to the electronic configuration of Co^4+^ ions, making the investigation of the temperature-dependent electrical resistivity of the mixture phase containing BCT-BaCoO_3_ essential for understanding its behavior. Electrical resistivity measurements were carried out on polycrystalline pellets of BCT-BaCoO_3_ obtained from the mixture precursor. The pellet used for Figure 4 had approximate dimensions of 1.0 × 0.8 × 1.0 mm^3^, with density estimated to be the theoretical value based on the measured mass and volume (Archimedes method). Four-probe contacts were made using Pt wires attached with silver epoxy; the geometry was carefully controlled to minimize contact resistance. We also measured additional pellets from independent syntheses and observed consistent temperature-dependent behavior, confirming reproducibility. Resistivity exhibits insulating behavior (Figure 4a). At room temperature, its zero-field resistivity, approximately 25 mΩ cm, is characteristic of poorly conducting oxides.^11^ Resistivity increases gradually as temperature decreases to 100 K, then a minor field-dependent feature appears near the FM ordering temperature, shown in Figure 4a inset. Should Co^3+^ ions also exist in the mixture phase containing BCT-BaCoO_3_ alongside Co^4+^ ions, Co–O–Co double exchange might induce a metallic state. However, we observed insulating behavior, with resistivity rising from 10^–1^ Ω cm at 300 K to nearly 10^5^ Ω cm at 20 K. Furthermore, the observed negligible magnetoresistance (MR) contrasts with the expected significant negative MR in double-exchange ferromagnets.^37^ The feature near the FM ordering temperature is more apparent upon examining the temperature derivative of electrical resistivity, as depicted in Figure 4b. This feature appears to be quenched under fields following the FM ordering, indicating a link to changes in the magnetic structure. A small anomaly is observed near the ferromagnetic transition at ∼107 K (Figure 4 *b*). This feature becomes less pronounced under applied fields up to 5 T, indicating that it is closely tied to changes in magnetic ordering. Such coupling between spin and charge degrees of freedom is consistent with the strong Co–O–Co superexchange interactions inferred from structural analysis.

**Figure 4:**
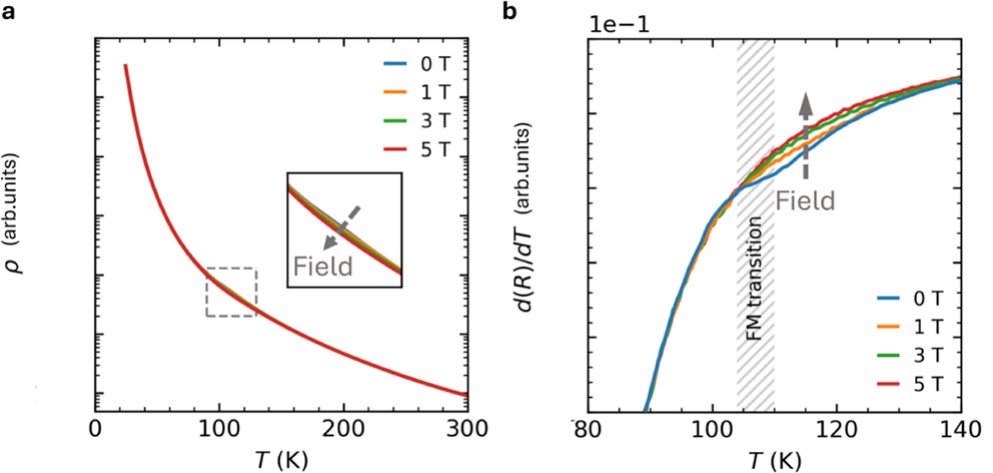
Field and temperature dependence of electrical resistivity in BCT-BaCoO_3_. a, Electrical resistivity as a function of temperature under an applied field up to 5 T. Inset: A detailed view within the gray dashed box highlights subtle differences across fields. b, Temperature derivative of electrical resistivity, showing a small feature quenched under fields near the FM ordering temperature region.

Figure 5 delineates the temperature ranges of various processes, including thermal activation of charge carriers below the FM transition temperature (65–110 K), and Mott variable-range hopping (VRH) in Region I (245–300 K) and II (165–210 K). In the range 65–110 K, the data follow an Arrhenius-type activated behavior with activation energy 356(5) K (*R* ^2^ = 0.9999); at higher temperatures, variable-range hopping (VRH) provides better fits, with *T*_0_ = 1.24(3) × 10^7^ K for 165–210 K and *T*_0_ = 1.88(5) × 10^7^ K for 245–300 K (*R* ^2^ > 0.9999 in both cases). These results are summarized in Table S3.^38^

**Figure 5:**
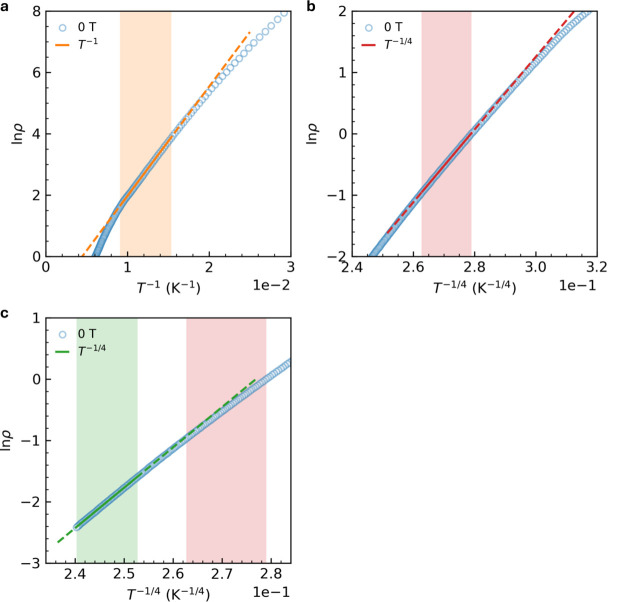
Temperature variations of the zero-field electrical resistance. a, ln ρ versus *T* ^–1^ plot. b–c, ln ρ versus *T* ^–1/4^ plot in Region I (green) and Region II (red). Temperature range of data fitting is indicated by color filling.

To purify BCT-BaCoO_3_, we attempted to obtain a purified 2H-BaCoO_3_ precursor. Figure S11 presents powder X-ray diffraction data for the purified 2H-BaCoO_3_ precursor and for the sample after high-pressure and high-temperature treatment. The fitted phases correspond to standard 2H-BaCoO_3_ from XRD analysis and to single-crystal XRD results of high-pressure synthesized pure 2H-BaCoO_3_. Figures S12 and S13 show magnetization, resistivity, and specific heat measurements. All results indicate possible degradation of the high-pressure phase at ambient pressure, with no metastable phases detected.

To elucidate the magnetic and electrical properties of tetragonal BCT-BaCoO_3_, we performed density functional theory (DFT) calculations. DFT suggests paramagnetic BaCoO_3_ exhibits metallic behavior with a d^7^ valence and a Kondo-like peak at the Fermi level, attributable to Co *t*_2g_ bands, as depicted in Figure 6a and b. Incorporating dynamical mean-field theory (DMFT) into DFT calculations, in contrast, reveals fluctuations between the d^5^ and d^6^ valences, as illustrated in Figure 6c–e. Additionally, *t*_2g_ orbitals experience a Mott transition, resulting in the opening of a very small gap (and a larger pseudogap) in the spectra, as shown in Figure 6c and d. *N* = 5 multiplets show minimal double occupancy, in contrast to *N* = 6 multiplets which necessitate some double occupancy, indicating a negligible preference for *t*_2g_ or *e*_g_ orbital occupation. For *N* = 6 multiplets, a slight preference exists for *e*_g_ orbitals due to crystal electric fields. This aligns with our experimental observations. Notably, electrons in both *N* = 5 and *N* = 6 multiplets fluctuate into spin-flipped states, as depicted in Figure 6f and g, despite strong Hund’s coupling.

**Figure 6:**
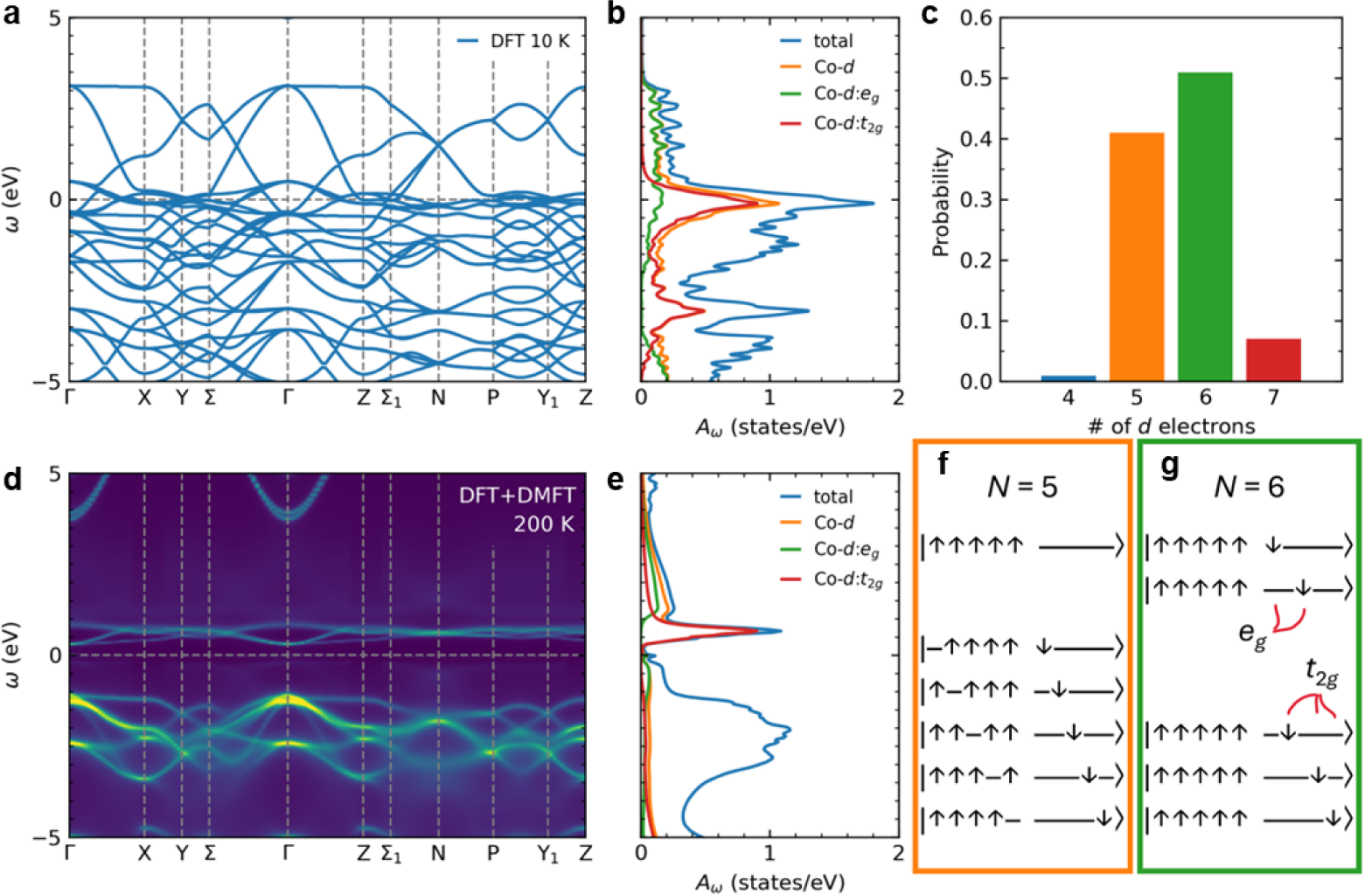
Electronic structure calculations of BCT-BaCoO_3_. a–d, Band structure and density of states analyzed using DFT, alongside angle-resolved and integrated spectral functions derived from DFT + DMFT. DFT suggests metallic behavior; conversely, DFT + DMFT reveals a minimal gap, attributed to the divergence of *t*_2g_ electron self-energy. Incoherent, localized *e*_2g_ states persist in the pseudogap just below the Fermi level, resulting in an enlarged pseudogap. e, DFT+DMFT impurity histograms show significant fluctuations in the d^6^ (and even d^7^) valence and substantial spin fluctuations, particularly into states which minimize double occupancy like those depicted in f and g. There is a slight preference to occupy *e*_g_ states when orbitals are doubly occupied as in the d^6^ multiplets.

## Conclusion

We report the synthesis of a tetragonal perovskite phase, BCT-BaCoO_3_, at 15 GPa and 1200 °C. HR-STEM analysis and powder X-ray diffraction reveal that BCT-BaCoO_3_ crystallizes in the *I*4/*mcm* (No. 140) space group, adopting a EuTiO_3_-type structure. No detectable oxygen vacancies were observed, consistent with the Co^4+^ oxidation state indicated by XPS measurements. Magnetization data show ferromagnetic ordering at approximately 107 K, attributable to BCT-BaCoO_3_. Above this temperature, the mixed-phase sample exhibits Curie–Weiss paramagnetic behavior, along with an LS–HS crossover upon cooling and a possible intermediate-spin state at higher temperatures. Electrical resistivity measurements indicate insulating behavior with weak magnetoresistance. DFT and DFT + DMFT calculations suggest that the insulating state originates from an orbitally selective transition sensitive to the nominal valence of the Co-d shell. Importantly, we attempted to purify BaCoO_3_ as a precursor for future measurements; however, the metastable BCT-BaCoO_3_ phase could not be preserved at ambient pressure in pure form. Significant atomic-site disorder was observed, indicating degradation of the high-pressure phase. This observation provides insight into how metastable phases can be stabilized at ambient pressure. We conclude that metastable BCT-BaCoO_3_ is stabilized at ambient pressure by embedding within a highly disordered mixture, exhibiting ferromagnetic insulating behavior below 107 K, and offering a promising route for discovering and stabilizing other high-pressure phases under ambient conditions.

## Supplementary Material



## Notes

SEM pictures, EDS analysis, powder X-ray diffraction measurements, additional magnetization and specific heat measurements, and related fitting (PDF)
