# Supplementary material for: Tetragonal BaCoO 3 : A Co 4+ Ferromagnetic Mott Insulator
Source: J Phys Chem C Nanomater Interfaces. 2025 Oct 23;129(44):19887–96. doi: 10.1021/acs.jpcc.5c03983 (PMC12598853; doi:10.1021/acs.jpcc.5c03983)
Supplement: Supplementary file 1 [file jp5c03983_si_001.pdf]

Supplementary Information

Tetragonal $BaCoO_3$: A Co^{4+} Ferromagnetic Mott Insulator

Mingyu Xu[1], Haozhe Wang[1], Krishna Prasad Koirala[2], Corey Melnick[3], Cheng Peng[1], Mario U. González-Rivas[4,5], Jiaqi Lu[6], Le Wang[2], Jessica Freese[5,7], Mark H. Engelhard[8], Yingge Du[2], Xianglin Ke[9], Robert J. Green[5,7], Alannah M. Hallas[4,5], Jie Li[6], Gabriel Kotliar[3,10], Weiwei Xie[1*]

1. Department of Chemistry, Michigan State University, East Lansing, MI 48824, USA
2. Physical and Computational Sciences Directorate, Pacific Northwest National Laboratory, Richland, WA 99354, USA
3. Condensed Matter Physics and Materials Science Department, Brookhaven National Laboratory, Upton, NY 11973, USA
4. Department of Physics & Astronomy, University of British Columbia, Vancouver, BC V6T 1Z1, Canada
5. Stewart Blusson Quantum Matter Institute, University of British Columbia, Vancouver, BC V6T 1Z4, Canada
6. Department of Earth and Environmental Sciences, University of Michigan, Ann Arbor, MI 48109, USA
7. Department of Physics and Engineering Physics, University of Saskatchewan, Saskatoon S7N 5E2, Saskatchewan, Canada
8. Energy and Environment Directorate, Pacific Northwest National Laboratory, Richland, WA 99354, USA
9. Department of Physics and Astronomy, Michigan State University, East Lansing, MI 48824, USA
10. Department of Physics and Astronomy, Rutgers University, Piscataway, NJ 08854, USA

[*]Corresponding authors: Dr. Weiwei Xie (xieweiwe@msu.edu)

Table of Contents

Experimental Methods……………………………………………………………………S3

Fig. S1 | Optical microscope images of BCT-$BaCoO_3$………….……………………S6

Fig. S2 | Chemical element distribution of BCT-$BaCoO_3$……………………….…...……S7

Fig. S3 | Spatial variation of Ba, Co, and O based on EDS analysis…………………………S9

Fig. S4 | PXRD on the sample prepared from the mix precursor ……………………..……S9

Fig. S5 | **Magnetization on the sample prepared from the mix precursor**………………….S10

Fig. S6 | Heat capacity on the sample prepared from the mix precursor………………….S11

Fig. S7 | Temperature-dependent magnetization in the range of 100–300 K………………S12

Fig. S8 | Field-dependent magnetization up to 7 T above 100 K……………………………S13

Fig. S9 | Electronic structure and chemical valence analysis revealed by XPS……………S15

Fig. S10 | PXRD on the sample prepared from the pure precursor ……………………..…S16

Fig. S11 | **Magnetization on the sample prepared from the pure precursor**………………S17

Fig. S12 | **Heat capacity on the sample prepared from the pure precursor**……………….S17

Table S1 | Spatial variation of Ba, Co, and O based on EDS analysis……..………………S18

Table S2 | Curie-Weiss fitting parameters of magnetic susceptibility………………………S19

Table S3 | Thermal activation fitting parameters of electrical resistance…………………..S20

Note S1 | Chemical valence analysis of BCT-$BaCoO_3$ revealed by XPS…………………..S21

Reference……………………………………………………………………………S22

Methods

High Pressure and High Temperature Synthesis. The synthesis was conducted using a 1000-ton multi-anvil apparatus (MA) at the University of Michigan. The starting material was synthesized at ambient pressure, which was prepared by thoroughly mixing the $BaCO_3$ and CoO in the atomic ratio of 1:1 and subsequently heating at 1100 °C for 72 hours before quenching.[1] The sample was kept at 110 °C overnight to remove the moisture before loading for high-pressure synthesis. Pure $BaCoO_3$($P6_3/mmc$) is prepared at 800°C for 72 hours before quenching. The COMPRES 10/5 cell assemblies were used in the synthesis.[2] The sample was loaded in a platinum capsule and kept at 15 GPa and 1200 °C for 3 hours before quenching to room temperature and then decompressed to ambient pressure. High-pressure and high-temperature synthesis were processed three times to get consistent results.

Phase Analysis. The phase identity and purity were examined using a Bruker Davinci powder X-ray diffractometer with Cu K_α radiation (λ = 1.5406 Å). Samples were ground using a mortar and pestle at room temperature, and then the powder was put on the vacuum grease-coated silicon puck. Room temperature measurements were carefully performed with a step size of 0.010° at a scan speed of 5.00 sec/step over a Bragg angle (2θ) range of 15-90°. The synthesis product was examined using the JEOL-7800FLV field emission SEM at the Robert B. Mitchell Electron Microbeam Analysis Lab (EMAL) of the University of Michigan, and the analyses confirmed chemical purity and homogeneity.

Structure and Chemical Composition Determination. The structural and chemical composition determination was conducted using high-angle annular dark field (HAADF) imaging and energy dispersive X-ray spectroscopy (EDS) inside a scanning transmission electron microscope (STEM). The TEM sample was prepared by using a dual beam Helios instrument, which combines focused ion beam (FIB) and scanning electron microscopy. First, a cross-sectional lamella was extracted from the polycrystalline sample using FIB milling. The lamella gradually thinned down to approximately 200 nm at 30 kV. Subsequently, the sample was further reduced to thickness to around 50 nm at 5 kV. The final polishing of the sample was carried out at 2 kV. For HAADF imaging, a Themis-Z STEM microscope equipped with an aberration corrector and a monochromator was used. An acceleration voltage of 300 kV and a prove current of approximately

30 pA were employed for both STEM imaging and EDS mapping. In HAADF imaging, a convergence angle of 30 mrad and collection angle of 60 to 180 mrad were used.

Chemical Valence State Analysis. X-ray photoelectron spectroscopy (XPS) measurements were performed using a Thermo Fisher NEXSA spectrometer with a 125 mm mean radius, full 180° hemispherical analyzer, and 128-channel detector. This system uses a focused monochromatic Al K_α X-ray (1486.7 eV) source for excitation and an electron emission angle of 60 degrees. The narrow scan spectra were collected using a pass-energy of 50 eV with a step size of 0.1 eV. For the Ag $3d_{5/2}$ line, these conditions produced a FWHM of 0.84 eV ± 0.02 eV. The binding energy (BE) scale is calibrated using the Cu $2p_{3/2}$ feature at 932.62 ± 0.05 eV and Au $4f_{7/2}$ at 83.96 ± 0.05 eV.

Physical Properties Measurement. Magnetization measurements were carried out using a Quantum Design MPMS 3 magnetometer and PPMS after demagnetization using SQUID and VSM. Sample are glue to the no-background quartz rod with GE varnish. Temperature dependent magnetization was measured over the temperature range of 2–300 K employing the zero-field cooled (ZFC) and field cooled (FC) protocols. Magnetic hysteresis loops were recorded with applied fields up to 7 T. Electrical resistivity measurements were conducted with four-probe methods using platinum wires on a polycrystalline sample of BCT-$BaCoO_3$ in the dimensions of 1.0 × 0.8 × 1.0 mm^3 with a Quantum Design physical property measurement system (PPMS) DynaCool.

Electronic Structure Calculation. We conduct all-electron density functional theory (DFT) and charge self-consistent DFT with dynamical mean field theory (DFT+DMFT) of BCT-$BaCoO_3$ using *Portobello*[3-5]. The DFT equations are solved within the generalized-gradient approximation (GGA) using the Perdew-Burke-Ernzerhof (PBE)[6] functional, neglecting the spin-orbit coupling. An 8 × 8 × 8 *k*-mesh and basis with RK of 8 are used for all calculations. The DMFT equations are used to treat correlations within the Co *d*-shell, where the off-diagonal elements in the Hamiltonian are truncated in order to avoid a sign problem during the solution of the quantum impurity problem. We use a spherically symmetric Slater-Condon interaction[7] with Hubbard U = 10 eV and Hund J = 1 eV to describe the interaction, and we use the fully localized limit double-counting with an electron occupancy of $N_0 = 5$, which correspond to the nominal d^5 valence.

X-ray Absorption Spectroscopy: X-ray absorption spectroscopy (XAS) experiments were carried out at the REIXS beamline of the Canadian Light Source.[8] The experiment was carried out at normal incidence, at several temperatures between 20 K and 300 K, spanning the different regions identified from the Curie-Weiss fit of the magnetic susceptibility. Total electron yield was obtained from by measuring the drain current from the sample. The samples were mounted on silver paint to improve thermal contact. Data collection was carried out between 750 eV and 830 eV, spanning the Co L2,3 and Ba M 4,5 resonances. Due to the overlap between the Co and Ba transitions, a $BaTiO_3$ thin film was used as a Ba^{2+} reference. The $BaTiO_3$ spectrum was used to remove the Ba M 4,5 white line from the $BaCoO_3$ sample.

Supplementary Figures

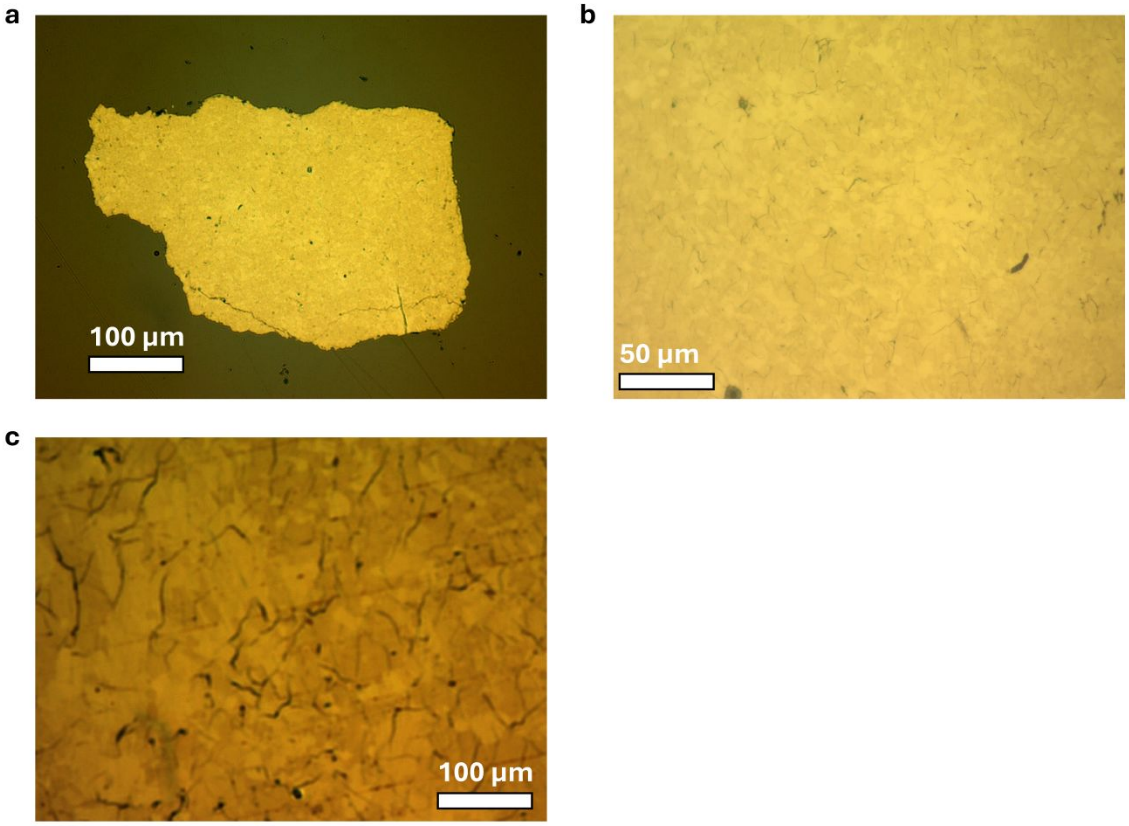

Fig. S1 | Optical microscope images of BCT-$BaCoO_3$. a, Overall view of polished BCT-$BaCoO_3$ with 5× magnification. **b,** Zoom-in view with 20× magnification. **c,** Zoom-in view in the contrast mode with 50× magnification.

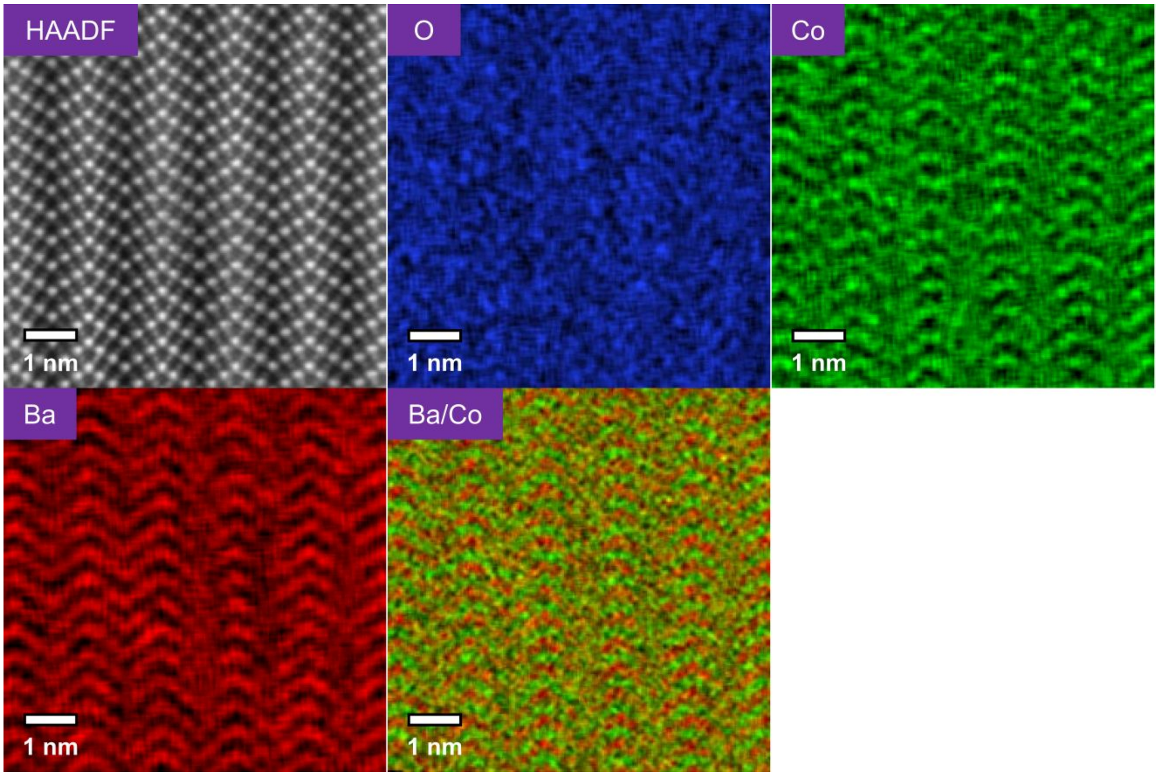

Fig. S2 | Chemical element distribution of BCT-$BaCoO_3$. HAADF-STEM image and EDS mapping images of O, Co, Ba and Ba/Co are presented respectively.

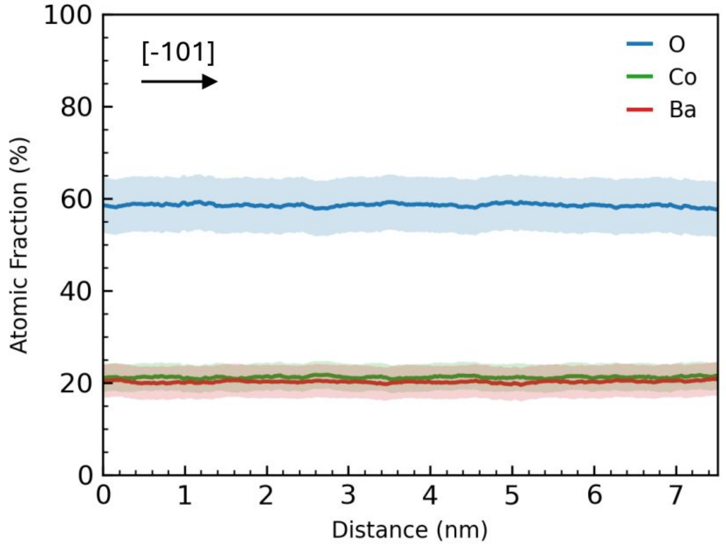

Fig. S3 | Spatial variation of Ba, Co, and O based on EDS analysis. The error bar is indicated by color filling.

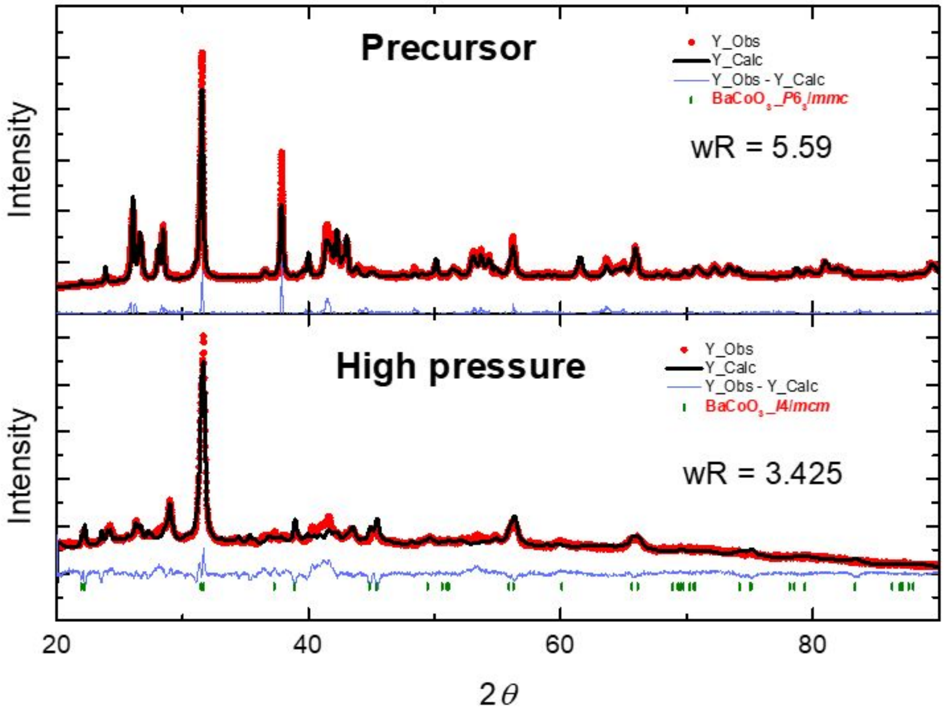

Fig. S4 | Powder X-ray diffraction measurements on the high-pressure, high-temperature synthesized sample with precursor synthesized at 1100 ℃ .

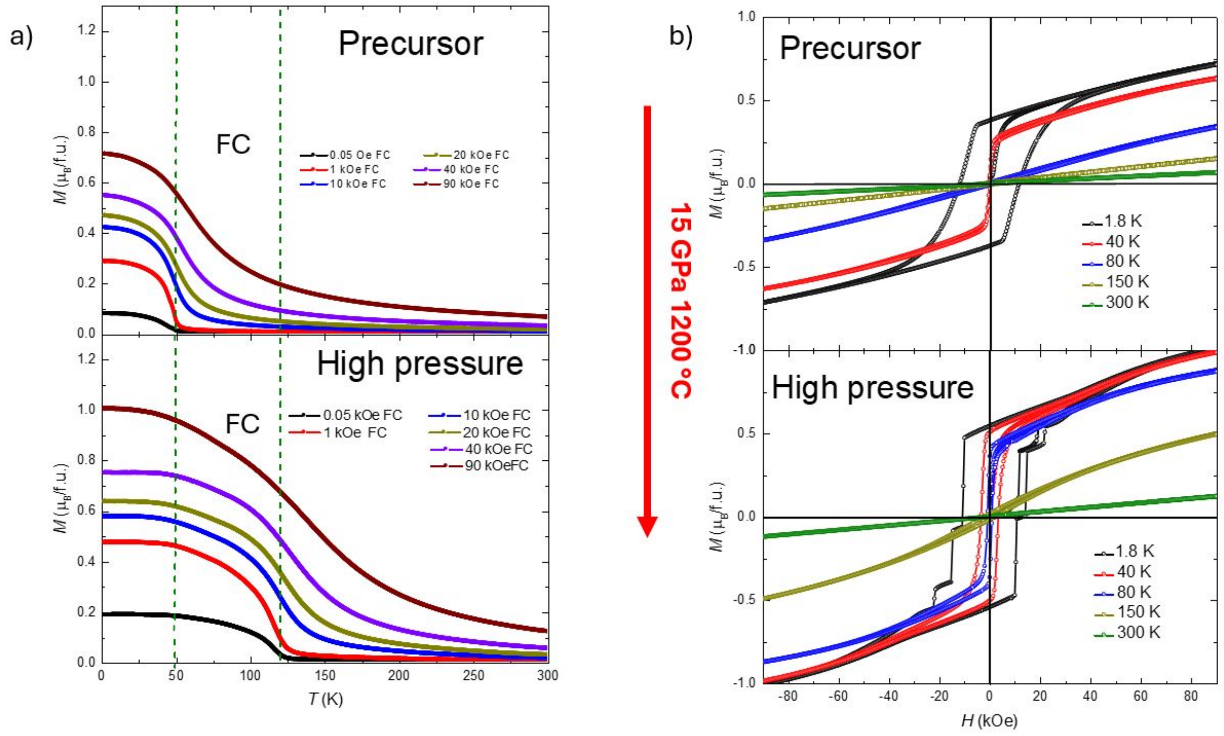

Fig. S5 | Temperature-dependent and field-dependent magnetization of the sample prepared from the mix-phase precursor synthesized at 1100℃. (a) Temperature-dependent magnetization of the sample prepared from a mixed-phase precursor synthesized at 1100 °C. The green dashed line indicates the change of ferromagnetic behavior. This repeat measurement was performed to confirm experimental consistency. (b) Field-dependent magnetization.

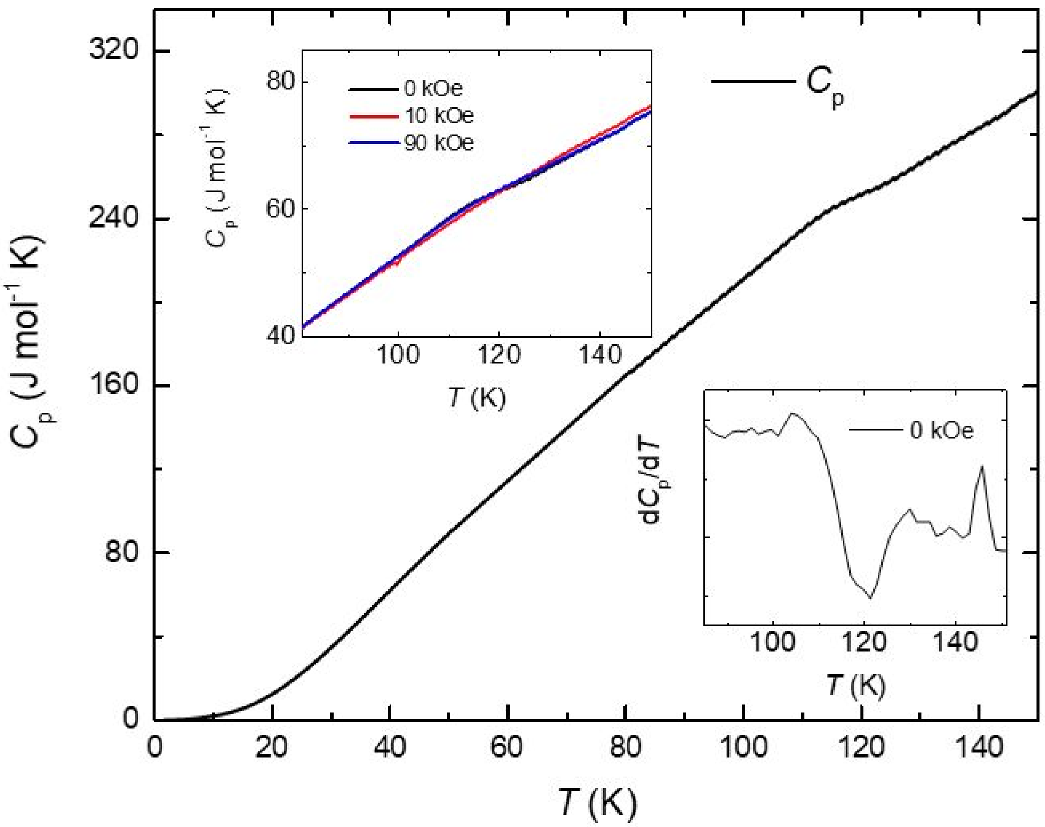

Fig. S6 | Heat capacity of the sample prepared from the mix-phase precursor synthesized at 1100℃. Insets show the temperature-dependent heat capacity at different fields and the derivative of heat capacity.

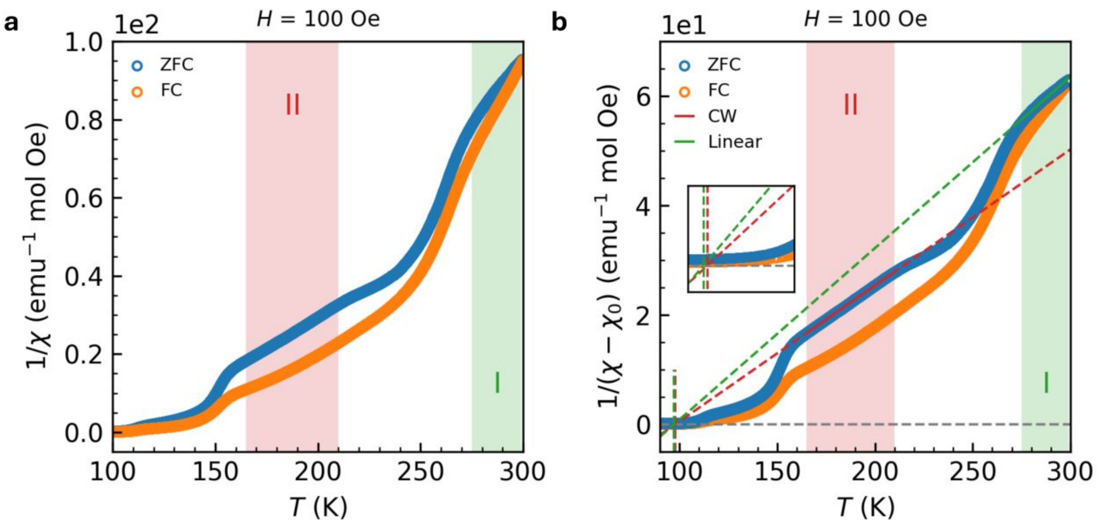

Fig. S7 | Temperature-dependent magnetization in the range of 100–300 K. a, Inverse magnetic susceptibility in temperature Regions I (green) and II (red). **b,** Magnetic susceptibility in temperature Region II fitted by Curie-Weiss law (red), in which Region I was linear fitted with the given χ_0 (green). Inset, Zoom-in plot near 100 K.

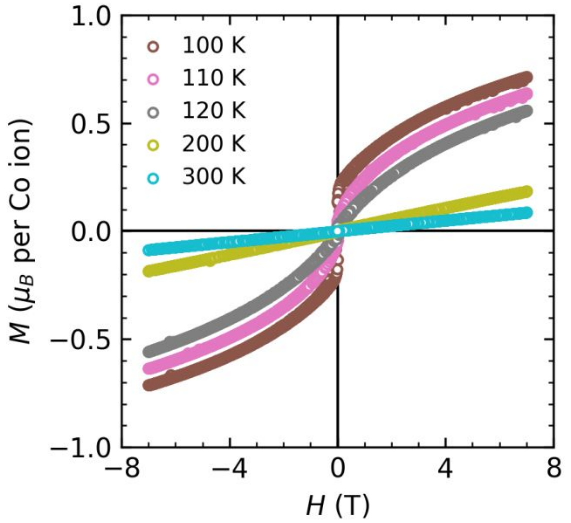

Fig. S8 | Field dependent magnetization of BCT-$BaCoO_3$ up to 7 T above 100 K. Linear response to fields at high temperatures confirms its paramagnetic behavior.

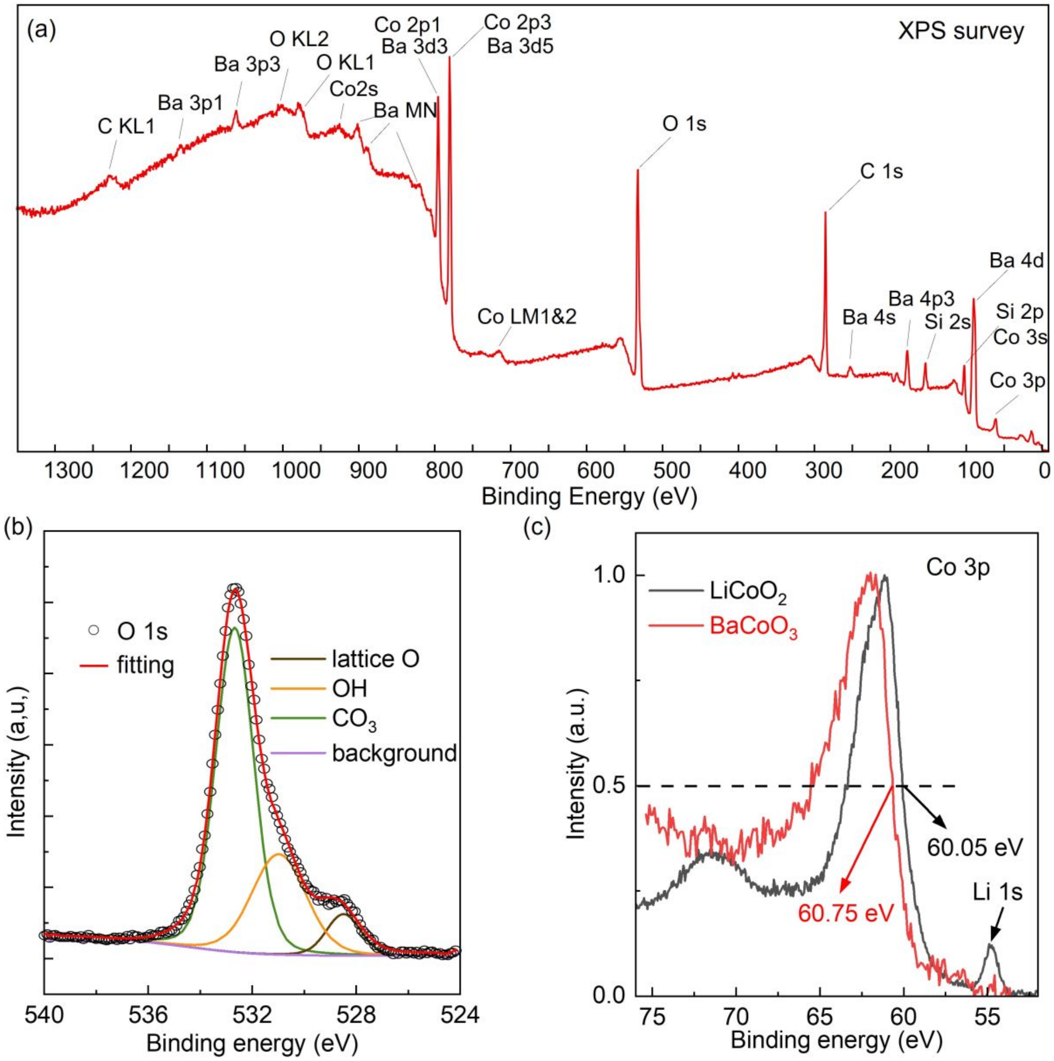

Fig. S9 | Electronic structure and chemical valence analysis revealed by XPS. a, Survey XPS spectra. **b-c,** O 1*s* and Co 3*p* core level XPS spectra.

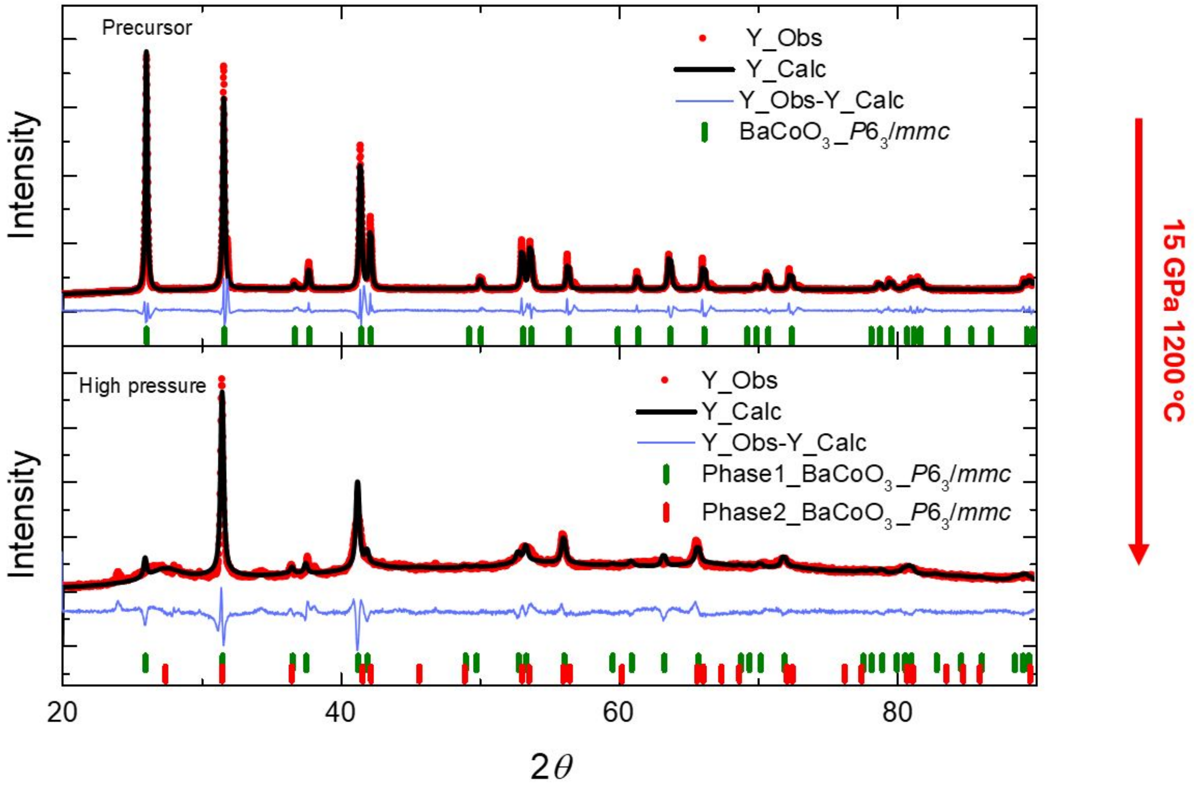

Fig. S10 | Powder X-ray diffraction measurements on the high-pressure, high-temperature synthesized sample with precursor synthesized at 800 ℃.

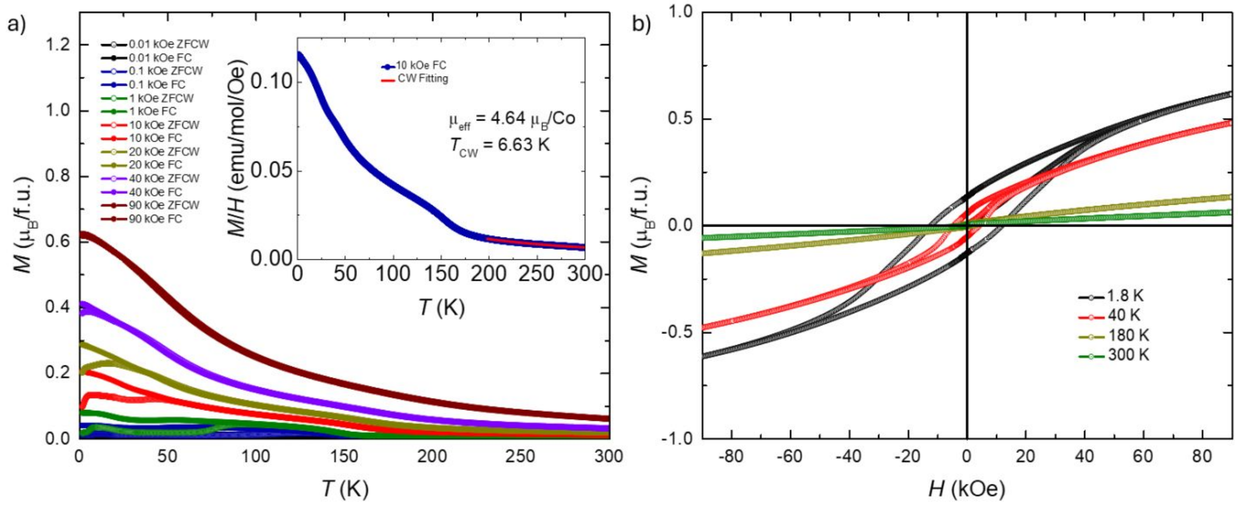

Fig. S11 | Temperature-dependent and field-dependent magnetization on the high-pressure, high-temperature synthesized sample with precursor synthesized at 800 ℃.

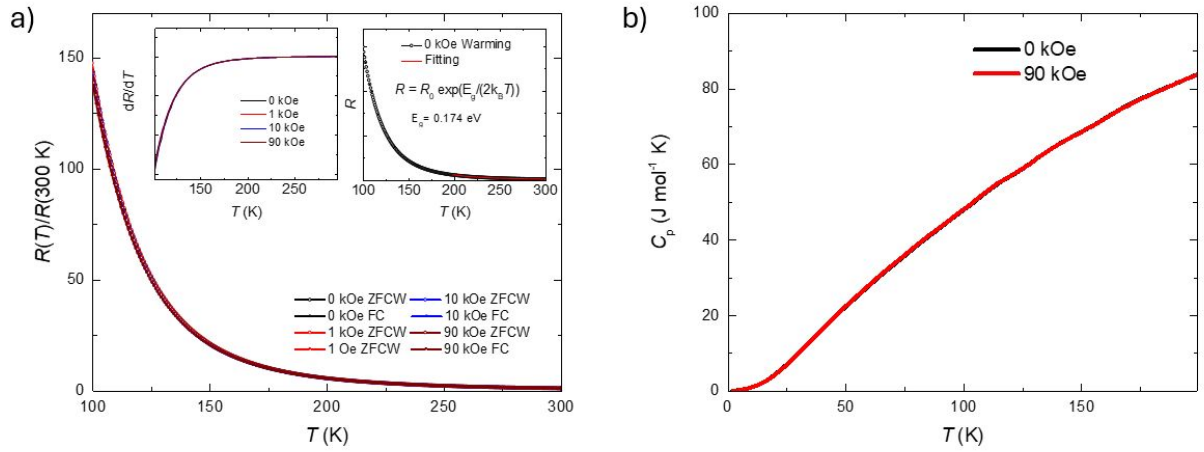

Fig. S12 | Resistance and heat capacity measurements on the high-pressure, high-temperature synthesized sample with precursor synthesized at 800 ℃.

Supplementary Tables

Table S1 | Spatial variation of Ba, Co, and O based on EDS analysis. The values are averaged of 0-7.5 nm along [-101] direction.

Element	Atomic Fraction / %	Atomic Fraction Error / %	Normalized Ratio
O	58.67	6.19	2.78
Co	21.14	3.60	1
Ba	20.09	3.04	0.95

Table S2 | Curie-Weiss fitting parameters of temperature dependent magnetic susceptibility. The equation is presented below. r^2 is the coefficient of determination.

$$\frac{1}{\chi - \chi_0} = \frac{T - \theta_{CW}}{C}$$

where θ_{cw} is the paramagnetic Curie temperature, χ_0 is the temperature independent susceptibility and C is the Curie constant.

Curie-Weiss fitting

Temperature Range / K	θ_{cw} / K	C / (emu K/mol-Oe)	μ_{eff} / μ_B	r^2
275–299	225.6(4)	0.3293(44)	1.62(1)	0.99984
165–210	97.8(2)	4.025(1)	5.67(1)	0.99997

Temperature Range / K	χ_0 / (emu/mol-Oe)
275–299	$6.037(36) \times 10^{-3}$
165–210	$-5.370(74) \times 10^{-3}$

Linear

Temperature Range / K	θ_{cw} / K	C / (emu K /mol-Oe)	μ_{eff} / μ_B	r^2
275–299	97.2	3.191	5.05	0.99509
165–210	120.2	2.229	4.22	0.99803

Table S3 | Thermal activation fitting parameters of electrical resistance. The equation is presented below. r^2 is the coefficient of determination.

$$\ln \rho = \ln \rho_0 + \left(\frac{T_0}{T}\right)^{\nu}$$

where ρ_0 is the residual resistivity, and T_0 is the characteristic temperature.

$\nu = 1$

Temperature Range / K	ρ_0 / (Ω cm^{-1})	T_0 / K	r^2
65–110	0.2034	356.4	0.99996

$\nu = 1/4$

Temperature Range / K	ρ_0 / (Ω cm^{-1})	T_0 / K	r^2
165–210	6.623×10^{-8}	1.235×10^{7}	0.99993
245–300	1.217×10^{-8}	1.877×10^{7}	1.00000

Supplementary Notes

Note S1 | Chemical valence analysis of BCT-$BaCoO_3$ revealed by XPS.

To investigate the Co valence state in t*I*-$BaCoO_3$, X-ray photoelectron spectroscopy (XPS) experiments were carried out. A survey scan XPS spectrum of t*I*-$BaCoO_3$, covering the binding energy range of 0–1350 eV, is presented in **Fig. S7a**. The spectrum exhibits all characteristic lines corresponding to Ba, Co, and O, indicating the presence of these elements in the t*I*-$BaCoO_3$ sample. Additionally, a minor signal from Si was observed, which is attributed to residual contamination from the crucible used during the sample synthesis process. The Ba and Co ratio was estimated by analyzing the Ba 4*p* and Co 3*p* regions, yielding a ratio that is close to 1.

The O 1*s* spectrum (**Fig. S7b**) was analyzed by fitting with three distinct components. The low binding energy feature (528.5 eV) corresponds well with values reported in literature for bulk lattice oxygen.[9-11] At binding energy of 531.0 eV, a feature is attributed to the presence of hydroxyl group (OH). The most intense peak at 532.7 eV is indicative of the existence of carbonate species, which is supported by the significant contribution of the C 1*s* peak observed in the survey scan. Due to strong overlap between Ba 3*d* and Co 2*p* regions, the Co valence state was determined using the Co 3*p* spectrum (**Fig. S7c**). A Co^{3+} reference sample of as-grown $LiCoO_2$ thin film on $SrTiO_3$ substrate was chosen for comparison.[12,13] To easily visualize the change in Co 3*p* line shape, we align these two Co 3*p* XPS spectra to place the corresponding O 1*s* (lattice O) peaks at 530.0 eV. Compared with the Co^{3+} reference, Co 3*p* feature of BCT-$BaCoO_3$ appeared broader and lacked an obvious satellite feature (~72 eV).[14] Moreover, a significant shift towards higher binding energy was observed in the Co 3*p* peak of t*I*-$BaCoO_3$. This shift, approximately 0.7 eV, indicates that the Co valence state in t*I*-$BaCoO_3$ is considerably higher than Co^{3+}, close to Co^{4+}.[15,16]

References

1. Gushee, B. E.; Katz, L.; Ward, R., The Preparation of a Barium Cobalt Oxide and other Phases with Similar Structures. *J. Am. Chem. Soc.* **1957**, *79* (21), 5601-5603.
2. Leinenweber, K. D.; Tyburczy, J. A.; Sharp, T. G.; Soignard, E.; Diedrich, T.; Petuskey, W. B.; Wang, Y.; Mosenfelder, J. L., Cell assemblies for reproducible multi-anvil experiments (the COMPRES assemblies). *Am. Mineral.* **2012**, *97* (2-3), 353-368.
3. Adler, R.; Melnick, C.; Kotliar, G., Portobello - Quantum embedding in correlated materials made accessible. *Comput. Phys. Commun.* **2024**, *294*, 108907.
4. Melnick, C.; Sémon, P.; Yu, K.; D'Imperio, N.; Tremblay, A.-M.; Kotliar, G., Accelerated impurity solver for DMFT and its diagrammatic extensions. *Comput. Phys. Commun.* **2021**, *267*, 108075.
5. Kutepov, A.; Haule, K.; Savrasov, S. Y.; Kotliar, G., Electronic structure of Pu and Am metals by self-consistent relativistic GW method. *Phys. Rev. B* **2012**, *85* (15), 155129.
6. Perdew, J. P.; Burke, K.; Ernzerhof, M., Generalized Gradient Approximation Made Simple. *Phys. Rev. Lett.* **1996**, *77* (18), 3865-3868.
7. Cheng, X.; Fabbri, E.; Nachtegaal, M.; Castelli, I. E.; El Kazzi, M.; Haumont, R.; Marzari, N.; Schmidt, T. J., Oxygen Evolution Reaction on $La_{1-x}Sr_xCoO_3$ Perovskites: A Combined Experimental and Theoretical Study of Their Structural, Electronic, and Electrochemical Properties. *Chem. Mater.* **2015**, *27* (22), 7662-7672.
8. Crumlin, E. J.; Mutoro, E.; Liu, Z.; Grass, M. E.; Biegalski, M. D.; Lee, Y.-L.; Morgan, D.; Christen, H. M.; Bluhm, H.; Shao-Horn, Y., Surface strontium enrichment on highly active perovskites for oxygen electrocatalysis in solid oxide fuel cells. *Energy Environ. Sci.* **2012**, *5* (3), 6081-6088.
9. Natile, M. M.; Ugel, E.; Maccato, C.; Glisenti, A., $LaCoO_3$: Effect of synthesis conditions on properties and reactivity. *Appl. Catal. B Environ.* **2007**, *72* (3), 351-362.
10. Shen, Z.; Qu, M.; Shi, J.; Oropeza, F. E.; de la Peña O'Shea, V. A.; Gorni, G.; Tian, C. M.; Hofmann, J. P.; Cheng, J.; Li, J.; Zhang, K. H. L., Correlating the electronic structure of perovskite $La_{1-x}Sr_xCoO_3$ with activity for the oxygen evolution reaction: The critical role of Co 3*d* hole state. *J. Energy Chem.* **2022**, *65*, 637-645.

11. Stoerzinger, K. A.; Wang, L.; Su, H.; Lee, K.-J.; Crumlin, E. J.; Du, Y., Influence of strain on $SrFeO_{3-\delta}$ oxidation, reduction, and water dissociation: Insights from ambient pressure X-ray photoelectron spectroscopy. *Appl. Surf. Sci.* **2020**, *527*, 146919.
12. Samarakoon, W.; Hu, J.; Song, M.; Bowden, M.; Lahiri, N.; Liu, J.; Wang, L.; Droubay, T.; Koirala, K.; Zhou, H.; Feng, Z.; Tao, J.; Du, Y., Direct Imaging of the Structural and Morphological Evolution of Epitaxial $LiCoO_2$ Films during Charge and Overcharge. *J. Phys. Chem. C* **2022**, *126* (37), 15882-15890.
13. Wang, L.; Yang, Z.; Samarakoon, W. S.; Zhou, Y.; Bowden, M. E.; Zhou, H.; Tao, J.; Zhu, Z.; Lahiri, N.; Droubay, T. C.; Lebens-Higgins, Z.; Yin, X.; Tang, C. S.; Feng, Z.; Piper, L. F. J.; Wee, A. T. S.; Chambers, S. A.; Du, Y., Spontaneous Lithiation of Binary Oxides during Epitaxial Growth on $LiCoO_2$. *Nano Lett.* **2022**, *22* (13), 5530-5537.
14. Alex, C.; Sarma, S. C.; Peter, S. C.; John, N. S., Competing Effect of Co^{3+} Reducibility and Oxygen-Deficient Defects Toward High Oxygen Evolution Activity in Co_3O_4 Systems in Alkaline Medium. *ACS Appl. Energy Mater.* **2020**, *3* (6), 5439-5447.
15. Dahéron, L.; Dedryvère, R.; Martinez, H.; Ménétrier, M.; Denage, C.; Delmas, C.; Gonbeau, D., Electron Transfer Mechanisms upon Lithium Deintercalation from $LiCoO_2$ to CoO_2 Investigated by XPS. *Chem. Mater.* **2008**, *20* (2), 583-590.
16. Wang, L.; Adiga, P.; Zhao, J.; Samarakoon, W. S.; Stoerzinger, K. A.; Spurgeon, S. R.; Matthews, B. E.; Bowden, M. E.; Sushko, P. V.; Kaspar, T. C.; Sterbinsky, G. E.; Heald, S. M.; Wang, H.; Wangoh, L. W.; Wu, J.; Guo, E.-J.; Qian, H.; Wang, J.; Varga, T.; Thevuthasan, S.; Feng, Z.; Yang, W.; Du, Y.; Chambers, S. A., Understanding the Electronic Structure Evolution of Epitaxial $LaNi_{1-x}Fe_xO_3$ Thin Films for Water Oxidation. *Nano Lett.* **2021**, *21* (19), 8324-8331.
